# Fine mapping linkage analysis identifies a novel susceptibility locus for myopia on chromosome 2q37 adjacent to but not overlapping MYP12

**Published:** 2009-04-10

**Authors:** Maria Schäche, Christine Y. Chen, Kelly Kathleen Pertile, Andrea Jane Richardson, Mohamed Dirani, Paul Mitchell, Paul Nigel Baird

**Affiliations:** 1 Centre for Eye Research Australia, University of Melbourne, Royal Victorian Eye & Ear Hospital, Melbourne, Australia; 2 Vision Cooperative Research Centre, Sydney, Australia; 3 Centre for Vision Research, Department of Ophthalmology, Westmead Millennium Institute, University of Sydney, Westmead, Australia

## Abstract

**Purpose:**

Myopia (shortsightedness) is one of the most common ocular conditions worldwide and results in blurred distance vision. It is a complex trait influenced by both genetic and environmental factors. We have previously reported linkage of myopia to a 13.01 cM region of chromosome 2q37 in three large multigenerational Australian families that initially overlapped with the known myopia locus, MYP12. The purpose of this study was to perform fine mapping of this region and identify single nucleotide polymorphisms (SNPs) associated with myopia.

**Methods:**

Fine mapping linkage analysis was performed on three multigenerational families with common myopia to refine the previously mapped critical interval. SNPs in the region were also genotyped to assess for association with myopia using an independent case-control cohort.

**Results:**

The disease interval was refined to a 1.83 cM region that is adjacent to rather than overlapping with the MYP12 locus. Subsequent sequencing of all known and hypothetical genes as well as an association study using an independent myopia case-control cohort showed suggestive but not statistically significant association to two intronic SNPs.

**Conclusions:**

We have identified a novel locus for common myopia on chromosome 2q37.

## Introduction

Shortsightedness is one of the most common eye conditions clinically manifesting as blurred distance vision. It results when light rays entering the eye are focused in front of rather than on the retina. Prevalence rates for myopia range from 20%–25% in Western countries to over 80% in the urbanized populations of Singapore, China, Japan, and Taiwan [1-6]. Myopia can be clinically defined using spherical equivalent (SphE) measurements that are quantitated using diopter (D) measurements. Individuals having readings of −0.5 D or less in both eyes are considered to be myopic [7]. Clinically, myopia can be classified as common (SphE ≤−0.5 to <−6 D) or high grade (SphE ≤−6 D). The more severe grades of myopia are associated with an increased risk of sight threatening complications such as glaucoma, retinal changes, and cataract [7,8]. Due to the high prevalence of myopia worldwide together with the increased risk of visual morbidity from associated complications, myopia is a significant public health problem. As a consequence, the search for risk factors involved in myopia is paramount if we are to gain insights into its pathogenesis and reduce its burden on health.

Myopia is a complex trait influenced by both genetic and environmental factors [9]. Environmental factors such as reading (near work) are known to influence the development of myopia but appear to account for only 12% of the observed phenotypic variance [10]. The remaining influences on myopia have been suggested to relate to genes as heritability studies indicate that between 50%–94% of population variance is accounted for by genetic factors [11-15]. In support of these heritability studies, familial correlation studies have shown that children with myopic parents have a four times greater risk of developing myopia than children with non-myopic parents [16]. In addition, genetic mapping studies have identified at least 18 chromosomal regions suspected of harboring a myopia gene (MYP1–MYP18). Of these regions, 11 have been implicated in high myopia (MYP1– MYP5, MYP11, MYP12, MYP13, MYP15, MYP16, MYP18) [17-28] and seven in common myopia (MYP6–MYP10, MYP14, MYP17) [29-31]. Five of these loci (MYP2, MYP3, MYP6, MYP10, MYP13) have been confirmed through replication studies in independent family studies [32-38]. Recently, we reported replication of the MYP12 locus using three multigenerational Australian families [39]. The MYP12 locus was initially reported as harboring a gene for high grade myopia whereas our replication study indicated that this locus may also harbor a gene for milder forms of myopia.

We now present fine mapping data for these three Australian myopia families that has refined the mapped myopia interval to a region on chromosome 2q37 that is adjacent to but not overlapping the MYP12 locus. Our findings suggest this is a novel locus for myopia that is distinct from MYP12. Furthermore, we have undertaken an independent association study to identify potential genetic variants that may be associated with myopia.

## Methods

### Subjects

The three families used in this study were recruited as part of the Genes In Myopia (GEM) Study (Melbourne, Australia), and details regarding their ocular examination have been previously reported [40]. These families had previously undergone a 10 cM genome-wide scan that identified a myopia susceptibility locus to chromosome 2q37 [39]. DNA from all consenting family members was collected from venous blood samples as previously described [41]. For the current study, we defined myopic individuals as family members with spherical equivalent refraction equal to or less than −0.5 diopter sphere (DS) in both eyes and controls as family members with measurements greater than −0.5 DS in both eyes [42-47].

For the case-control association study, we used unrelated individuals also recruited through the GEM Study [40], the Blue Mountains Eye Study (BMES) [48], and the Melbourne Visual Impairment Project (VIP) [49]. Individuals with a history of eye diseases such as keratoconus, age related macular degeneration (AMD), or a history of genetic disorders known to predispose to myopia such as Stickler syndrome or Marfan syndrome were excluded. Using the same definition of what constituted myopia as used in the linkage study, we analyzed 300 myopic and 291 control individuals in a case-control cohort.

This study adhered to the tenets of the Declaration of Helsinki and was approved by the Royal Victoria Eye and Ear Hospital (Melbourne, Australia) and Westmead Hospital at the University of Sydney (Sydney, Australia). All individuals were of Caucasian descent and provided informed written consent before commencement of the study.

### Fine mapping linkage analysis

For fine mapping linkage analysis, the starting interval was defined by critical recombination events observed in the three families during the genome-wide linkage analysis [39]. This interval spanned markers D2S396 and D2S338 on chromosome 2q37. For fine mapping, polymorphic microsatellite markers spanning this mapped interval were chosen from the deCODE genetic maps [50]. Genotyping was undertaken by the Australian Genome Research Facility (AGRF; Melbourne, Australia) using a model 377 automated DNA sequencer (Applied Biosystems, Melbourne, Australia). All available family members were included in the genotyping. Genotype error checking was performed using PedManager version 0.9. Multipoint parametric and non-parametric linkage analyses were performed using MERLIN (version 1.1.2) [51]. In the case of parametric linkage analysis, two autosomal dominant models were used with phenocopy rates of 0.1 (model 1) or 0.2 (model 2), a penetrance of 0.9, and disease allele frequency of 0.0133. The choice of these models was based on those described by Chen et al. [15] in the initial linkage paper for these families. Haplotypes were generated using MERLIN (version 1.1.2) [51] and visualized using HaploPainter (version 027beta) [52].

### Candidate gene sequencing in the fine mapped region

Identification of all known and hypothetical genes in the refined interval was achieved by mining Ensembl [53], National Centre for Biotechnology Information (NCBI), and University of California Santa Cruz (UCSC) Genome Browser [54]. Bidirectional DNA sequencing was undertaken for all predicted and known exonic regions of these genes and at least 20 bp into each adjacent intron. DNA sequencing was performed in six myopic individuals (two randomly chosen from each of the three families) as well as six unrelated controls (married-in individuals). Primer design, template amplification, and DNA sequencing was performed by the Australian Genome Research Facility (Brisbane, Australia). Sequencing data was visualized using Chromas Lite (version 2.01) and ClustalX (version 2.0) [55] to identify any variations from the reference sequence deposited in the Ensembl database (reference sequence) [53]. The reference sequence for the known single nucleotide polymorphisms (SNPs) was defined by the common allele for the CEU (Utah residents with ancestry from northern and western Europe) population.

Following candidate gene screening, a subset of SNPs of interest were identified and chosen for further analysis in an independent case-control association study. SNPs that were present in only one of the six samples were not present in each of the three families or were present in an equal number of myopic individuals and controls were excluded from subsequent genotype analysis.

### Case-control association study

The subset of SNPs that fulfilled the above criteria was genotyped in an independent cohort of unrelated individuals of Caucasian descent. A total of 300 individuals with myopia and 291 controls were genotyped at the Australian Genome Research Facility (Brisbane, Australia) using the MassArray platform (Sequenom Inc., San Diego, CA) and matrix assisted laser desorption/ionization-time of flight (MALDI-TOF) analysis (Sequenom, San Diego, CA). To provide more extensive coverage of intronic and intergenic regions, additional SNPs that were evenly spaced across the entire refined linkage interval were identified using HapMap (Build #36) [56] and also genotyped in the case-control cohort.

Genotyping data was assessed for deviations from the Hardy–Weinberg equilibrium using PLINK [57]. Any SNPs not passing this test (p<0.05 in the controls) were noted and excluded from further analysis. Association tests were also performed using PLINK, and adjustments for multiple testing were made using the Bonferroni correction. Prospective power calculations were performed using Episheet.

## Results

### Fine mapping linkage analysis

**Figure 1:**
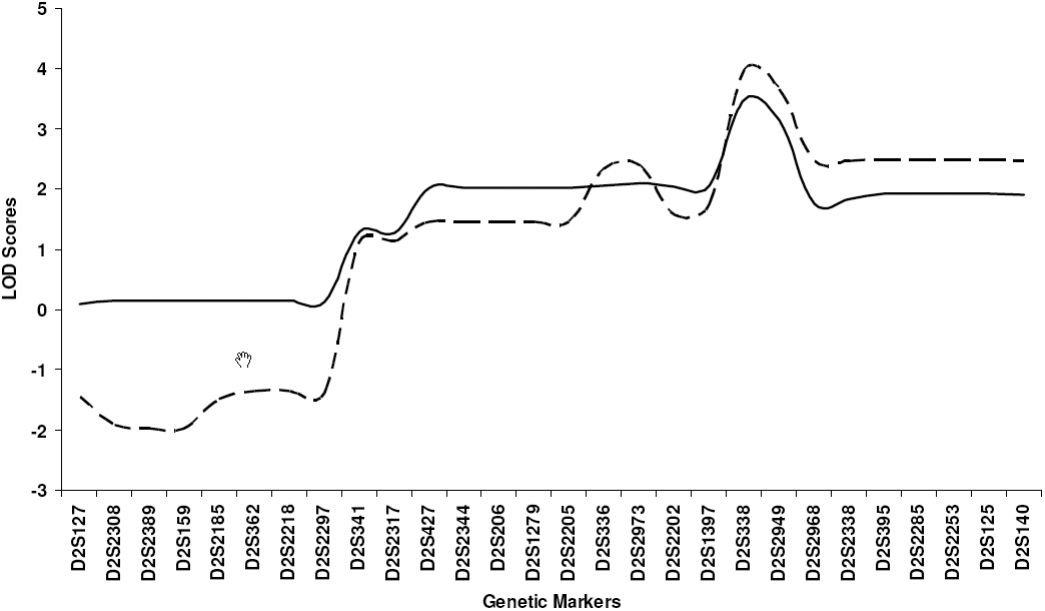
LOD Scores for the fine mapping linkage analysis on chromosome 2q37. Parametric (solid line) and nonparametric (dashed line) LOD scores are shown for the three GEM families (GEM0046, GEM0251, and GEM0206).

**Figure 2:**
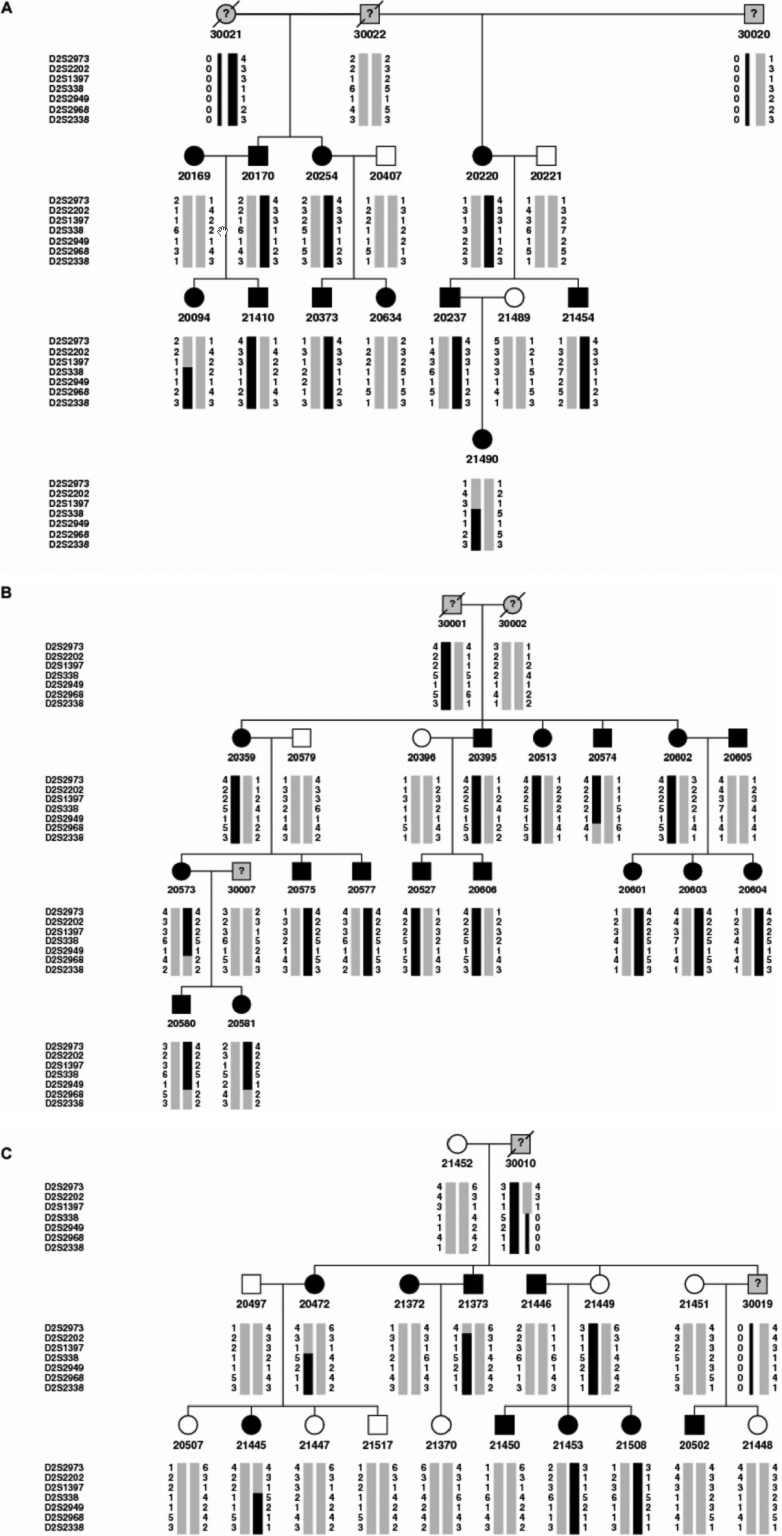
Haplotype analysis in the chromosome 2q37 linkage region for the three GEM families. Haplotype analysis in the chromosome 2q37 linkage region is shown for GEM0206 (**A**), GEM0046 (**B**), and GEM0251 (**C**).

**Figure 3:**
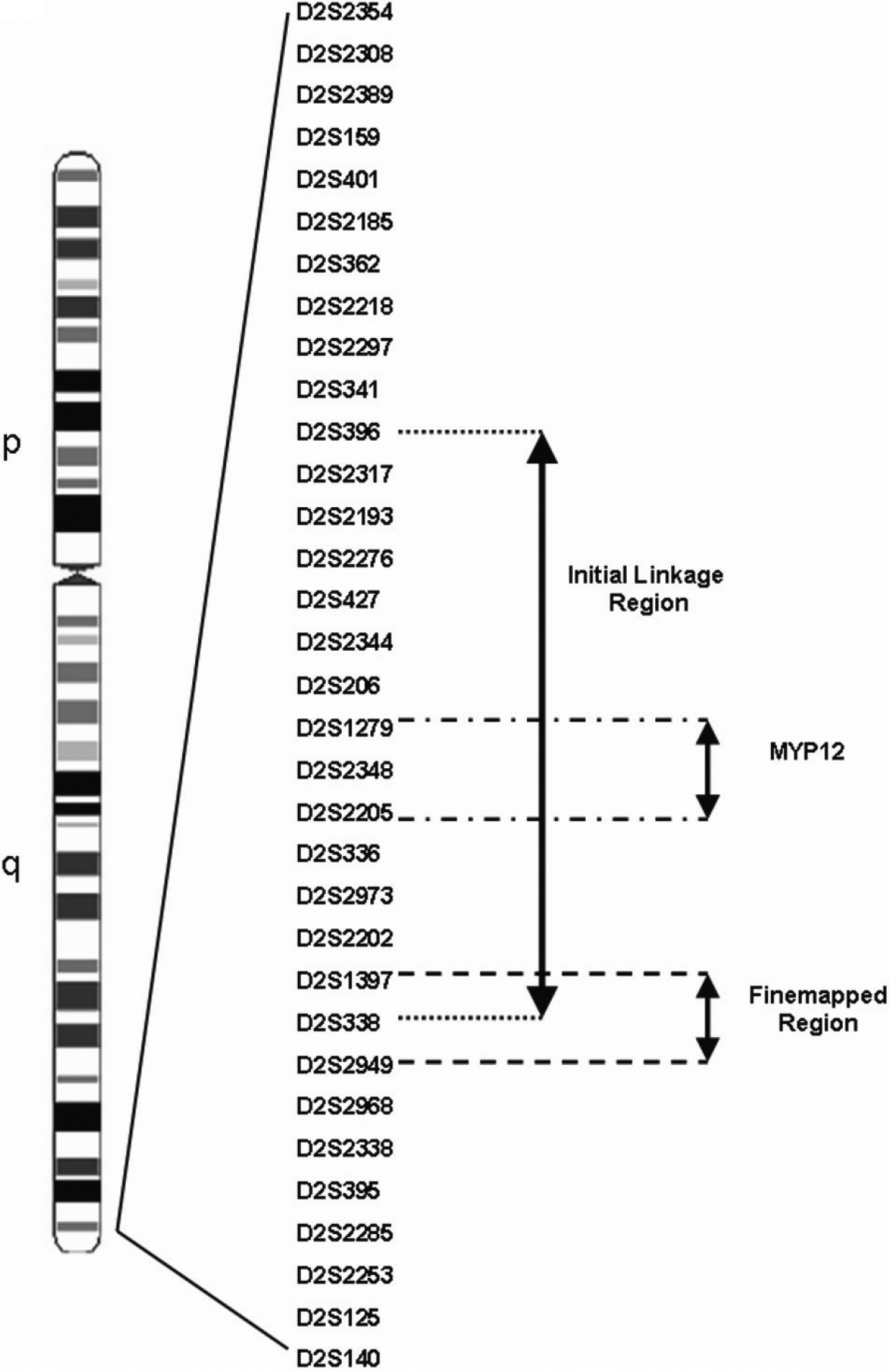
Ideogram of human chromosome 2 showing the location of the newly fine mapped region relative to the original linkage region and MYP12. Fine mapping linkage analysis now clearly indicates that the region of interest is adjacent to but does not overlap MYP12.

Previous linkage mapping in three myopia families (GEMF0046, GEMF0206, and GEMF0251) identified a 13.01 cM region at 2q37.1 that overlapped with the high myopia locus, MYP12 [39]. A total of 37 myopic and 14 control individuals (including three additional myopic individuals that were not previously available) from these three families were used for fine mapping. Fine mapping of this region resulted in a peak parametric and non-parametric LOD score of 3.97 and 3.48, respectively, at marker D2S338 (Figure 1). This was in agreement with the location of the peak LOD scores reported from our initial genome-wide scan. Haplotype analysis of this region and adjacent regions (6.02 cM proximal and 11.06 cM distal) using 30 polymorphic microsatellite markers at an average spacing of 1.2 cM in the families allowed significant narrowing of the myopia disease gene region (Figure 2). A critical recombination event in individuals 20573, 20580, and 20581 in GEMF0206 (Figure 2A) defined the distal end of the interval to marker D2S2968. A critical recombination event in individuals 20094 and 21490 from GEMF0046 (Figure 2B) and in individuals 21445 and 20472 from GEMF0251 (Figure 2C) defined the proximal end of the refined critical interval to marker D2S1397. The refined interval was identified as a 1.83 cM region on chromosome 2q37.2-2q37.3 that was distal to but did not overlap with the adjacent MYP12 locus (Figure 3).

### Candidate gene sequencing

To identify a potential causative myopia gene or variant within the refined linkage interval, we identified all known and hypothetical genes in this 1.83 cM interval. A total of eight hypothetical (*BX647589*, *AK00798*, *AK056246*, *AK023507*, *LOC728074*, *LOC728087*, *LOC728067, Q9UFF6*) and six known (ankyrin repeat and SOCS box-containing [*ASB18*], centaurin gamma 2 [*CENTG2*], COP9 constitutive photomorphogenic homolog subunit 8 [Arabidopsis; *COPS8*], chemokine (C-X-C) motif receptor 7 [*CXCR7*], IQ motif containing with AAA domain [*IQCA*], and gastrulation brain homeobox 2 [*GBX2*]) genes were identified.

DNA sequencing was undertaken on all 67 exons and exon-intron boundaries in the known and hypothetical genes that were localized to the refined linkage interval. The intron-exon structure for the hypothetical genes was defined by aligning predicted mRNA sequences with genomic DNA using the BLAST algorithm. The exons were divided into 109 amplicons and sequenced bidirectionally in six myopic individuals from the three GEM families and six controls (no myopia). Overall sequencing success was 94% with seven amplicons located in genes *CXCR7* (exon 1 and 2), *IQCA* (exon 17), and *GBX2* (exon 1) proving difficult to sequence. From the successfully sequenced amplicons, a total of 77 SNPs were identified where at least one individual had a genotype that varied from the Ensembl reference sequence. Through the use of our exclusion criteria listed under Methods, this list of SNPs was narrowed to a total of 38. To allow for complete SNP coverage of the refined interval, we also identified all the known HapMap (Build 23a) SNPs located in the difficult to sequence amplicons and added these to our list of candidate SNPs. This brought the total number of SNPs from the hypothetical and known genes to 39.

In addition to the 39 SNPs in the known and hypothetical genes, information from HapMap was used to identify additional intronic and intergenic SNPs in the refined interval. Using this approach, 120 additional SNPs across the entire refined interval as well as the region 561 kb proximal and 835 kb distal to the critical recombination events were chosen. These were selected to ensure they were evenly spaced and covered the entire region. There was an approximate spacing of 4–55 kb (average 51 kb) between each SNP.

### Case-control association study

A total of 159 (39+120) SNPs were genotyped in an independent case-control cohort consisting of 300 myopic and 291 control individuals from Australia. Power calculations were undertaken and suggested that a cohort of this size will have 80% power to detect an odds ratio of 1.78. The average genotyping success rate for these SNPs was 98.1%. A total of eight SNPs (5.0%) were found not to be in Hardy–Weinberg equilibrium and so were excluded from further analysis. An additional 14 SNPs (8.8%) were not polymorphic in this cohort, and they were also excluded from further analysis leaving 137 SNPs for the case-control association analysis. The 22 excluded SNPs were scattered throughout the linked region with no bias for one gene or genomic segment. All p values from the case-control association study for the remaining 137 SNPs underwent a Bonferroni correction. Using a conservative significance level of 5×10^−4^, we observed no statistically significant association to the genotyped SNPs [58].

## Discussion

Using a fine mapping linkage based approach, we have been able to identify a novel locus for myopia on chromosome 2q37. Initially, this region was described as overlapping with the known high grade myopia locus, MYP12. However, further fine mapping and haplotype analyses have enabled us to better refine this region to a smaller 1.83 cM segment on chromosome 2q37. Hence, the locus harboring the causative myopia gene in these GEM families is novel and distinct from the MYP12 locus, indicating a degree of genetic heterogeneity in this region of chromosome 2. Detailed SNP analysis and DNA sequencing of all known and hypothetical genes in the refined interval provided no evidence of a causative variant in the coding region of these genes. The best evidence for a causative variant in the region was provided by the two intronic SNPs of rs1986830 and rs4663724, but these only showed suggestive rather than statistically significant association.

Despite strong evidence for a hereditary component influencing myopia, identification of causative genes or DNA variants has so far proven difficult to achieve. To date, five genes, transforming growth factor beta induced factor homeobox 1 (*TGIF*) [59], paired box 6 (*PAX6*) [60], collagen, type 1, alpha 1 (*COL1A1*) [61], hepatocyte growth factor (*HGF*) [62], and uromodulin-like 1 (*UMODL1*) [27], have been positively associated with high grade myopia and collagen, type 2, alpha 1 (*COL2A1*) [63] with common myopia. Of these six candidates, *TGIF* and *COL1A1* can be excluded as candidates as subsequent replication studies have been negative [64-67]. *COL2A1*, *HGF*, and *UMODL1* have each been reported in single studies and await replication. The final gene, *PAX6*, has been positively associated with high grade myopia in two independent studies, but results have been negative for common myopia [29,63,68,69]. Hence, to date, *PAX6, HGF*, and *UMODL1* remain the strongest candidates for high grade myopia and collagen, type 2, alpha 1 (*COL2A1*) for common myopia.

The methodological approach that we have taken to gene identification, namely linkage analysis and DNA sequencing, mirrors the approach that has been used in many other studies mapping myopia loci [70-73]. Unfortunately, these studies have also failed to find a causative variant in the coding region of candidate genes. Given that myopia is a complex disease, it is possible that causative variants are located in intron or flanking regulatory regions as has been demonstrated for other complex diseases such as breast cancer, type 2 diabetes, and chronic kidney disease [74-76]. More detailed assessment of non-coding regions in these linked regions may provide more clues as to the genetic drivers of myopia. SNPs located in the non-coding sequence may affect gene/protein expression indirectly by affecting gene regulation and hence may be important drivers of the disease process. Given this, we extended our assessment of the candidate region to include intronic and intergenic SNPs spanning the linkage interval.

Our study has identified a relatively small linkage region on the long arm of chromosome 2 that represents a novel locus for common myopia. Further analysis of this region failed to convincingly identify genetic variants associated with myopia. However, there are a total of 1,048 known SNPs in the refined linkage interval, and we cannot rule out the possibility that additional SNPs in this region are associated with myopia. There are also several other issues that should be considered when interpreting our findings. One could argue that the cohort we used for the case-control study was underpowered to detect all variants that might be positively associated with myopia. While this is a possibility, the variants that are most likely to be missed are those that have small effect sizes. Although such variants may still contribute to myopia, they are likely to be only minor players. Furthermore, it is also important to be able to assess segregation of SNPs in the original GEM families used in the linkage analysis. However, the relatively small number of families used for this study would make segregation analysis statistically underpowered, and consequently, reliable data cannot be generated. We were aware of this study limitation from the onset and as a consequence, opted instead to validate SNPs in an independent case-control cohort to replicate the initial linkage result.

The findings presented here do not represent the conclusion of this study but do provide ongoing data for further investigation into the exact genetic causes of myopia. Further work needs to be undertaken to extend these findings to ensure complete coverage of this region. SNP genotyping also needs to be confirmed in a larger case-control cohort and also replicated in additional cohorts of both Caucasian and different ethnicities. Clearly, much more work is needed to further elucidate the underlying genetic influences on the development of myopia.
